# Supplementary material for: Exposure to Particulate PAHs on Potential Genotoxicity and Cancer Risk among School Children Living Near the Petrochemical Industry
Source: Int J Environ Res Public Health. 2021 Mar 4;18(5):2575. doi: 10.3390/ijerph18052575 (PMC7967639; doi:10.3390/ijerph18052575)
Supplement: Supplementary file 1 [file ijerph-18-02575-s001.pdf]

SUPPLEMENTARY MATERIALS

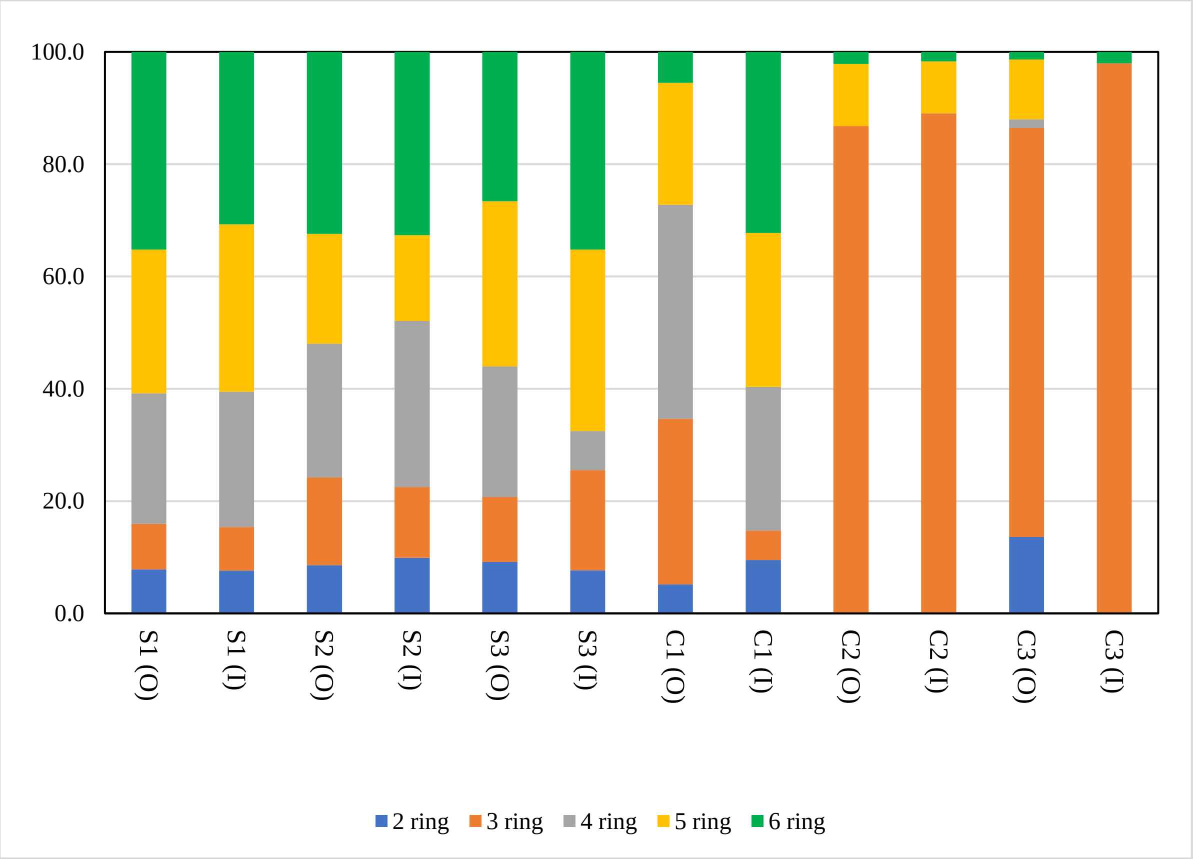

Figure S1: Percentage distribution of outdoor and indoor PAHs according to the number of rings

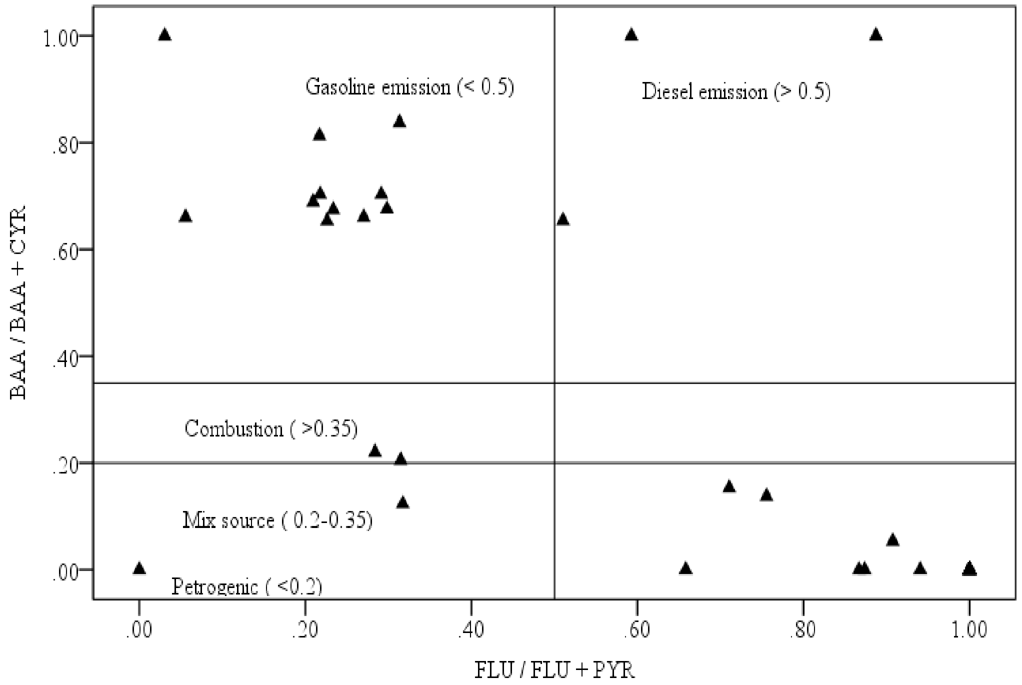

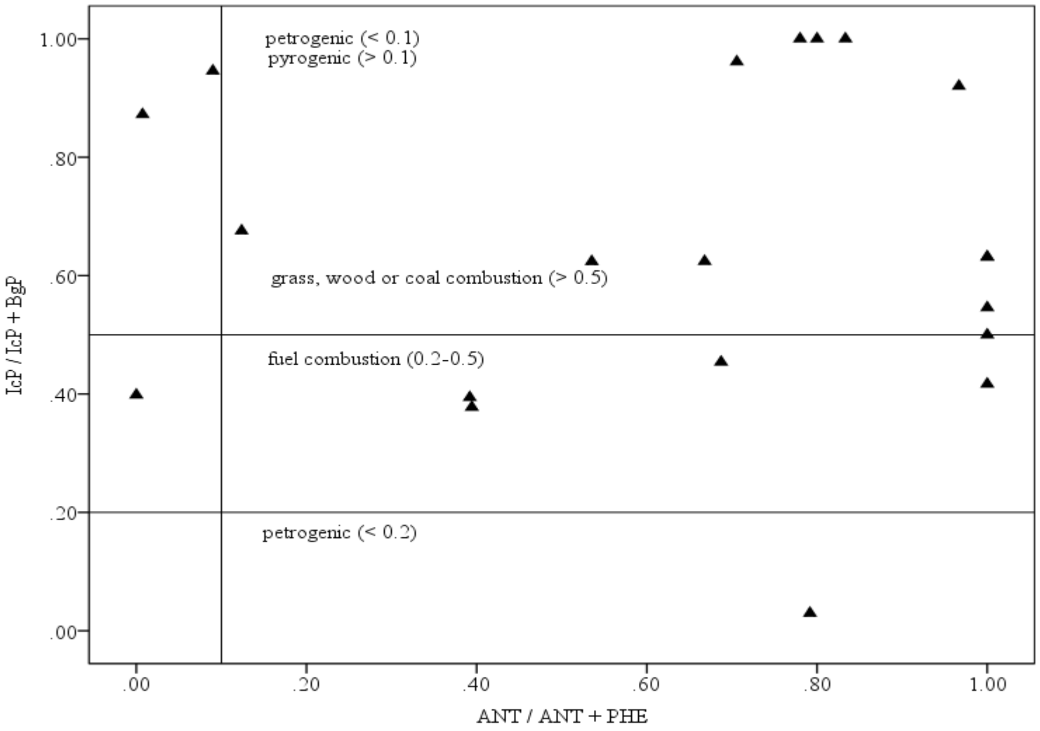

Figure S2: Source diagnostic ratio

Table S1: Descriptive information on the school's location

School	Coordinates	Number of students	Distance from the main road (m)	Distance from the industry (km)
S1	4°30'23.4" N 103°26'27.6"E	40	155.5	5.6
S2	4°31'04.6"N 103°26'41.2"E	33	31.4	4.4
S3	4°36'31.1"N 103°26'10.9"E	40	263.3	3.7
C1	4°45'10.8"N 103°11'18.3"E	40	805.30	35.96
C2	4°46'05.7"N 103°24'46.0"E	36	805.49	20.85
C3	4°24'23.8"N 103°23'46.7"E	28	42.91	20.66

Table S2 : Correlation coefficient and LOD of 16 priority PAHs

Congener	Correlation coefficient, R^2	LOD (ng m^{-3})
NAP	0.990	0.01
ACY	0.999	0.01
ACP	0.999	0.01
FLU	1.000	0.86
PHE	0.998	0.03
ANT	0.999	0.10
FLA	0.811	0.01
PYR	0.997	0.01
BaA	0.999	0.01
BkF	0.998	0.01
BbF	0.998	0.01
CYR	0.999	0.01
BaP	0.995	0.20
IcP	0.969	0.02
DbA	0.994	0.01
BgP	0.999	0.01

Table S3: Distribution of PAHs species in $PM_{2.5}$ samples

School	S1		S2		S3		C1		C2		C3	
Location	Outdoor	Indoor	Outdoor	Indoor	Outdoor	Indoor	Outdoor	Indoor	Outdoor	Indoor	Outdoor	Indoor
NAP	5.07 ± 4.26	4.80 ± 3.55	5.82 ± 3.01	5.45 ± 5.37	5.63 ± 4.53	3.39 ± 2.93	1.81 ± 0.00	1.24 ± 0.00	LOD	LOD	0.82 ± 0.00	LOD
FLU	3.30 ± 2.66	4.05 ± 2.07	5.35 ± 4.14	3.81 ± 3.12	4.50 ± 4.37	3.82 ± 3.49	6.84 ± 0.00	LOD	4.88 ± 0.33	4.11 ± 0.74	4.28 ± 1.03	3.86 ± 0.94
PHE	0.92 ± 0.70	0.86 ± 0.27	2.26 ± 2.82	1.98 ± 1.48	1.09 ± 0.52	1.72 ± 1.35	3.17 ± 0.00	0.30 ± 0.24	0.12 ± 0.00	0.02 ± 0.00	0.10 ± 0.07	0.09 ± 0.00
ANT	1.02 ± 0.87	LOD	2.95 ± 1.70	1.13 ± 0.98	1.56 ± 0.00	2.36 ± 0.98	0.32 ± 0.00	0.39 ± 0.00	0.15 ± 0.19	0.01 ± 0.00	LOD	0.16 ± 0.16
FLA	0.35 ± 0.00	LOD	0.40 ± 0.00	0.64 ± 0.00	LOD	LOD	LOD	LOD	LOD	LOD	LOD	LOD
PYR	9.31 ± 8.66	8.20 ± 7.86	12.98 ± 12.85	12.40 ± 9.13	11.57 ± 9.99	1.20 ± 0.49	13.00 ± 0.00	LOD	LOD	LOD	0.09 ± 0.00	LOD
BaA	3.70 ± 2.24	2.37 ± 1.82	1.21 ± 1.68	2.06 ± 2.08	1.78 ± 1.89	0.34 ± 0.35	0.25 ± 0.00	3.07 ± 1.60	LOD	LOD	LOD	LOD
BkF	1.03 ± 1.44	LOD	0.27 ± 0.19	1.00 ± 0.00	0.57 ± 0.65	0.31 ± 0.00	LOD	LOD	LOD	LOD	LOD	LOD
BbF	4.46 ± 2.68	3.55 ± 1.90	2.69 ± 1.79	2.66 ± 3.41	3.01 ± 1.24	2.31 ± 1.94	2.22 ± 0.00	0.84 ± 1.18	0.08 ± 0.00	LOD	0.34 ± 0.00	LOD
CYR	1.68 ± 1.06	1.12 ± 0.49	1.58 ± 0.82	1.15 ± 1.17	0.95 ± 0.74	1.56 ± 0.72	0.12 ± 0.00	0.28 ± 0.00	LOD	LOD	LOD	LOD
BaP	11.05 ± 5.07	14.45 ± 7.09	10.27 ± 2.15	4.74 ± 2.72	14.55 ± 5.16	11.67 ± 3.75	5.40 ± 7.53	2.75 ± 2.48	0.57 ± 0.00	0.43 ± 0.01	0.30 ± 0.00	LOD
IcP	9.12 ± 4.94	7.58 ± 2.03	8.83 ± 4.75	2.46 ± 2.12	6.59 ± 2.52	6.70 ± 2.84	0.99 ± 1.37	0.97 ± 1.25	0.04 ± 0.00	0.02 ± 0.01	0.03 ± 0.01	0.04 ± 0.04
DbA	6.65 ± 3.70	4.40 ± 2.02	4.81 ± 3.55	5.15 ± 4.78	1.99 ± 2.21	3.19 ± 2.28	0.87 ± 0.73	1.12 ± 1.52	0.03 ± 0.01	0.04 ± 0.02	0.03 ± 0.01	0.02 ± 0.00
BgP	6.99 ± 6.56	11.84 ± 4.85	8.31 ± 10.39	10.34± 11.40	7.81 ± 5.92	5.70 ± 7.30	0.07 ± 0.08	2.13 ± 0.66	0.06 ± 0.05	0.02 ± 0.00	0.02 ± 0.00	0.02 ± 0.00
Total PAHs	64.64 ± 44.85	63.22 ±33.95	67.72 ± 49.84	54.97 ±48.94	61.60 ± 39.74	44.27 ± 28.40	35.06 ± 9.71	13.09 ± 8.93	5.93 ± 0.59	4.65 ± 0.77	6.01 ± 1.19	4.21 ± 1.14

LOD: below limit of detection

Table S4: Toxic equivalent concentration for PAH compounds

	S1	S2	S3	C1	C2	C3
NAP	0.005	0.006	0.005	0.002	0.000	0.000
FLU	0.004	0.005	0.004	0.003	0.004	0.004
PHE	0.001	0.002	0.001	0.002	0.000	0.000
ANT	0.014	0.020	0.020	0.004	0.001	0.004
FLA	0.000	0.001	0.000	0.000	0.000	0.000
PYR	0.009	0.013	0.006	0.007	0.000	0.000
BaA	0.303	0.163	0.106	0.166	0.000	0.000
BkF	0.051	0.064	0.044	0.000	0.000	0.000
BbF	0.400	0.268	0.266	0.153	0.004	0.017
CYR	0.014	0.014	0.013	0.002	0.000	0.000
BaP	12.749	7.504	13.110	4.073	0.499	0.151
IcP	0.835	0.564	0.664	0.098	0.003	0.004
DbA	5.525	4.978	2.589	0.995	0.034	0.028
BgP	0.094	0.093	0.068	0.011	0.000	0.000
ΣTEQ	20.005	13.693	16.896	5.515	0.546	0.208

Table S5: The mean comparison tail moment (parameter of DNA damage) among children

School	N	Tail Moment	p-value
S1	25	25.56 ± 5.98	<0.001*
S2	23	31.89 ± 11.28	
S3	37	26.59 ± 7.53	
C1	48	20.43 ± 5.34	
C2	36	21.76 ± 4.75	
C3	36	20.78 ± 4.22	

ANOVA, *p*-value is significant at level 0.001

Table S6 : Relationship between tail moment with PAHs exposure and other risk factors

Variables	B	SE	β	*p*-value	95% CI	R^2
Total PAHs indoor[a]	0.082	0.018	0.291	<0.001**	0.047, 0.118	0.085
Carcinogen PAHs indoor[a]	0.174	0.036	0.307	<0.001**	0.103, 0.244	0.094
Non-carcinogen PAHs indoor[a]	0.l32	0.034	0.251	<0.001**	0.065, 0.198	0.063
Total PAHs outdoor[a]	0.056	0.013	0.266	<0.001**	0.029, 0.082	0.071
Carcinogen PAHs outdoor[a]	0.119	0.025	0.307	<0.001**	0.071, 0.167	0.094
Non-carcinogen PAHs outdoor[a]	0.083	0.028	0.194	0.003*	0.023, 0.137	0.038
Log 1-OHP[a]	0.254	2.984	0.010	0.932	-5.691, 6.199	0.932
Gender[b]	0.949	0.861	0.073	0.271	-0.748, 2.646	0.005
Age[a]	1.467	0.593	0.162	0.014*	0.299, 2.636	0.026
BMI[a]	0.248	0.118	0.144	0.037*	0.015, 0.480	0.021
ETS[b]	-0.579	0.868	-0.045	0.506	-2.290, 1.132	0.002
Grilled Diet[b]	0.639	0.870	0.049	0.463	-1.075	0.002
Supplement intake[b]	-1.145	0.923	-0.083	0.216	-2.964, 0.675	0.007
Mosquito coil[b]	1.750	1.078	0.107	0.106	-0.375, 3.874	0.012
Open burning[b]	-2.50	0.876	-0.187	0.005*	-4.227, - 0.774	0.035

Simple linear regression (method enter)
[a] = continuous variable, [b] = categorical variable
B = Unstandardized coefficient, SE = Standard Error
β = Regression coefficient
* *p*-value significant < 0.05, ** *p*-value significant < 0.001
R^2 = regression, 95% CI = 95% Confidence Interval
